# Bacterial Peptidoglycan Stimulates Adipocyte Lipolysis via NOD1

**DOI:** 10.1371/journal.pone.0097675

**Published:** 2014-05-14

**Authors:** Wendy Chi, Dyda Dao, Trevor C. Lau, Brandyn D. Henriksbo, Joseph F. Cavallari, Kevin P. Foley, Jonathan D. Schertzer

**Affiliations:** Department of Biochemistry and Biomedical Sciences, and Department of Pediatrics, McMaster University, Hamilton, Ontario, Canada; Tohoku University, Japan

## Abstract

Obesity is associated with inflammation that can drive metabolic defects such as hyperlipidemia and insulin resistance. Specific metabolites can contribute to inflammation, but nutrient intake and obesity are also associated with altered bacterial load in metabolic tissues (i.e. metabolic endotoxemia). These bacterial cues can contribute to obesity-induced inflammation. The specific bacterial components and host receptors that underpin altered metabolic responses are emerging. We previously showed that Nucleotide-binding oligomerization domain-containing protein 1 (NOD1) activation with bacterial peptidoglycan (PGN) caused insulin resistance in mice. We now show that PGN induces cell-autonomous lipolysis in adipocytes via NOD1. Specific bacterial PGN motifs stimulated lipolysis in white adipose tissue (WAT) explants from WT, but not NOD1^−/−^ mice. NOD1-activating PGN stimulated mitogen activated protein kinases (MAPK),protein kinase A (PKA), and NF-κB in 3T3-L1 adipocytes. The NOD1-mediated lipolysis response was partially reduced by inhibition of ERK1/2 or PKA alone, but not c-Jun N-terminal kinase (JNK). NOD1-stimulated lipolysis was partially dependent on NF-κB and was completely suppressed by inhibiting ERK1/2 and PKA simultaneously or hormone sensitive lipase (HSL). Our results demonstrate that bacterial PGN stimulates lipolysis in adipocytes by engaging a stress kinase, PKA, NF-κB-dependent lipolytic program. Bacterial NOD1 activation is positioned as a component of metabolic endotoxemia that can contribute to hyperlipidemia, systemic inflammation and insulin resistance by acting directly on adipocytes.

## Introduction

Obesity is associated with inflammation, which underpins defective metabolic and endocrine responses such as insulin resistance [1]–[3]. Adipose tissue expansion during obesity coincides with augmented inflammation, dysregulation of cytokines derived from adipose tissue (i.e. adipokines), and impaired insulin-mediated suppression of lipolysis. These changes in adipose tissue can contribute to ectopic lipid deposition and insulin resistance in the skeletal muscle and liver, the primary sites of post-prandial glucose disposal and glucose production [4]. In fact, lipolysis itself can promote inflammation in adipose tissue [5], setting up the potential for a vicious cycle of inflammation, insulin resistance and aberrant lipid metabolism. Understanding the triggers and host mediators of low-grade inflammation during obesity may provide new therapeutic strategies for metabolic disease. The integration of nutrient and pathogen sensing systems has prompted investigation of pattern recognition receptors (PRRs) in obesity-induced inflammation. PRRs have been proposed to propagate inflammatory cues from nutrient overload relevant to obesity. Saturated fat engaging PRRs may represent a form of a host-pathogen interaction by causing proinflammatory responses via Toll-like receptor (TLR)4, protein kinase R (PKR) and NOD-like receptor family, pyrin domain containing 3 (NLRP3) [6]–[9]. However, lipid-laden diets and obesity also induce alterations in circulating bacterial factors that contribute to PRR-mediated inflammation [10]–[12]. The etiology and metabolic effects of such bacterial cues are emerging.

Obesity and even a single meal containing fat have been associated with increased systemic bacterial components that are well-established ligands for PRRs [10], [13]–[15]. The exact cause of metabolic endotoxemia is not yet clear, but obesity has been associated with alteration in gut hormones and permeability providing the opportunity for components from the gut microbiota to contribute to increases in systemic factors that could activate PRRs [16]. This metabolic endotoxemia contributes to obesity-induced inflammation and insulin resistance [10]. Bacterial lipopolysaccharide (LPS) and TLR4 have been implicated in metabolic endotoxemia, but inputs from other bacterial components that interact with alternate PRRs are ill-defined. Gut microbiota derived bacterial peptidoglycan (PGN) can penetrate to internal sites, prime systemic innate immune responses and augment inflammation [17]. Nucleotide oligomerization domain (NOD)1 and NOD2 are an integral part of the mammalian repertoire that responds to bacterial PGN and are intracellular sensors that induce cytokine/defensin responses upon recognition of specific PGN motifs. NOD1 detects D-glutamyl-meso-diaminopimelic acid (meso-DAP)-containing PGN motif found mainly in Gram-negative bacteria. NOD2 detects muramyl dipeptide (MDP) PGN motif that is commonly more abundant in Gram-positive bacterial strains [18], [19].

We have shown that NOD1/2-double knockout mice are protected from high fat diet-induced obesity and insulin intolerance and that meso-DAP containing PGN causes inflammation and profound whole body insulin resistance via NOD1 [20]. We now sought to determine if bacterial PGN sensed by NOD1 or NOD2 could alter other metabolic defects seen during obesity. We and others have shown that NOD1 is expressed in adipocytes and that NOD1 activation causes proinflammatory responses in adipose tissue and adipocytes [20]–[23]. It is important to understand if bacterial PGN causes lipolysis through NOD1 or NOD2 because elevated circulating lipids and ectopic lipid deposition can promote insulin resistance in the liver and muscle [24]–[26].

Other bacterial factors have been implicated in lipid metabolism. For example, LPS acting on TLR4 can stimulate lipolysis in adipocytes [27]. We hypothesized that NOD1-activating PGN would also stimulate adipocyte lipolysis. Hormones such as epinephrine cause lipolysis through a G protein-coupled receptor-cAMP-protein kinase A (PKA) pathway. LPS and other inflammatory mediators such as tumor necrosis factor alpha (TNFα) activate ERK-dependent lipolysis [27], [28]. Both PKA and ERK pathways converge on lipases, such as hormone sensitive lipase (HSL) that regulate adipocyte lipolysis [29]. We sought to determine if PGN-mediated lipolysis was mediated through MAPKs (such as ERK) or PKA-mediated pathways. We found that NOD1-activating PGN stimulates adipocyte cell-autonomous HSL-mediated lipolyic program that engages ERK, PKA, and NF-κB.

## Results

### Bacterial PGN motifs cause adipose tissue lipolysis via NOD1

**Figure 1:**
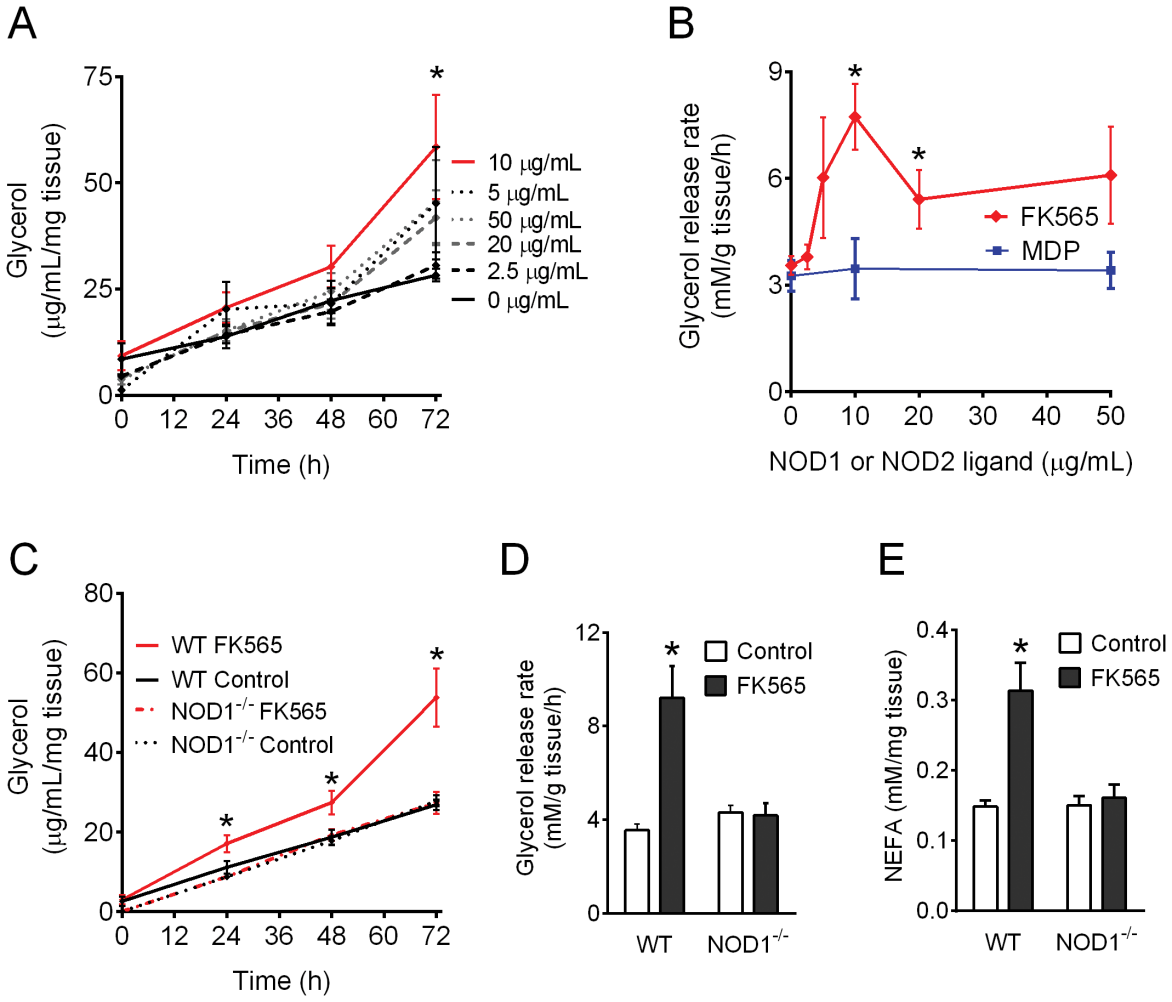
Bacterial PGN stimulates lipolysis via NOD1. Gonadal WAT explants from WT mice were incubated with various doses of FK565 and glycerol concentration in the media determined at 0, 24, 48 and 72(A). Glycerol release rate over 72 h was calculated in response to various doses of FK565 (NOD1 ligand) or MDP (NOD2 ligand) in WAT explants from WT mice (B). Gonadal WAT explants from WT or NOD1^−/−^ mice were incubated without (Control) or with FK565 (10 µg/mL) and glycerol concentration in the media determined at 0, 24, 48 and 72 h (C). Glycerol release rate over 72 h was calculated in explants from WT or NOD1^−/−^ mice, which were incubated with or without FK565 (D). Gonadal WAT explants from WT or NOD1^−/−^ mice were incubated with FK565 (10 µg/mL) and NEFA concentration in the media was determined at 72 h (E). n = 6–12 explants per condition, values are mean ± SEM. *Significantly different from the WT control condition or 0 µg/mL (i.e. no FK565) at a given time point.

Murine NOD1 preferentially detects diaminopimelic acid-type muramyl tetrapeptides of PGN 30]. Hence, we used FK565 (heptanoyl-γ-D-glutamyl-L-*meso* –diamino-pimelyl-D-alanine), a superior NOD1 ligand in murine cells compared to tripeptide meso-DAP containing PGN motifs such as iE-DAP or Tri-DAP. Our results show that 10 µg/mL of FK565 increased glycerol released into the media from WAT explants from WT mice (P<0.05, Fig 1A). Calculation of the glycerol release rate showed that a dose of 10-20 µg/mL of FK565 increased the glycerol release rate (a marker of lipolysis) in WAT explants from WT mice (P<0.05, Fig 1B). In contrast, the NOD2 ligand, MDP, did not alter glycerol release rate in WAT explants from WT mice (Fig 1B). Importantly, FK565 (10 µg/mL) increased glycerol concentration in the media at 24, 48 and 72 h in WAT explants from WT, but not NOD1^−/−^ mice (P<0.05, Fig 1C). Hence, FK565 increased the glycerol release rate from adipose tissue, an effect that was completely dependent on NOD1 (P<0.05, Fig 1D). Indices of NOD1-mediated lipolysis were not limited to increased glycerol release, since FK565 also increased the release of non-esterified fatty acids (NEFA) in WAT explants from WT, but not NOD1^−/−^ mice (P<0.05, Fig 1E). Adipose tissue contains many cell types (including immune cells) in addition to adipocytes. Hence, we next examined whether NOD1-activating PGN caused lipolysis directly in adipocytes.

### NOD1-activating PGN causes cell-autonomous lipolysis in adipocytes

**Figure 2:**
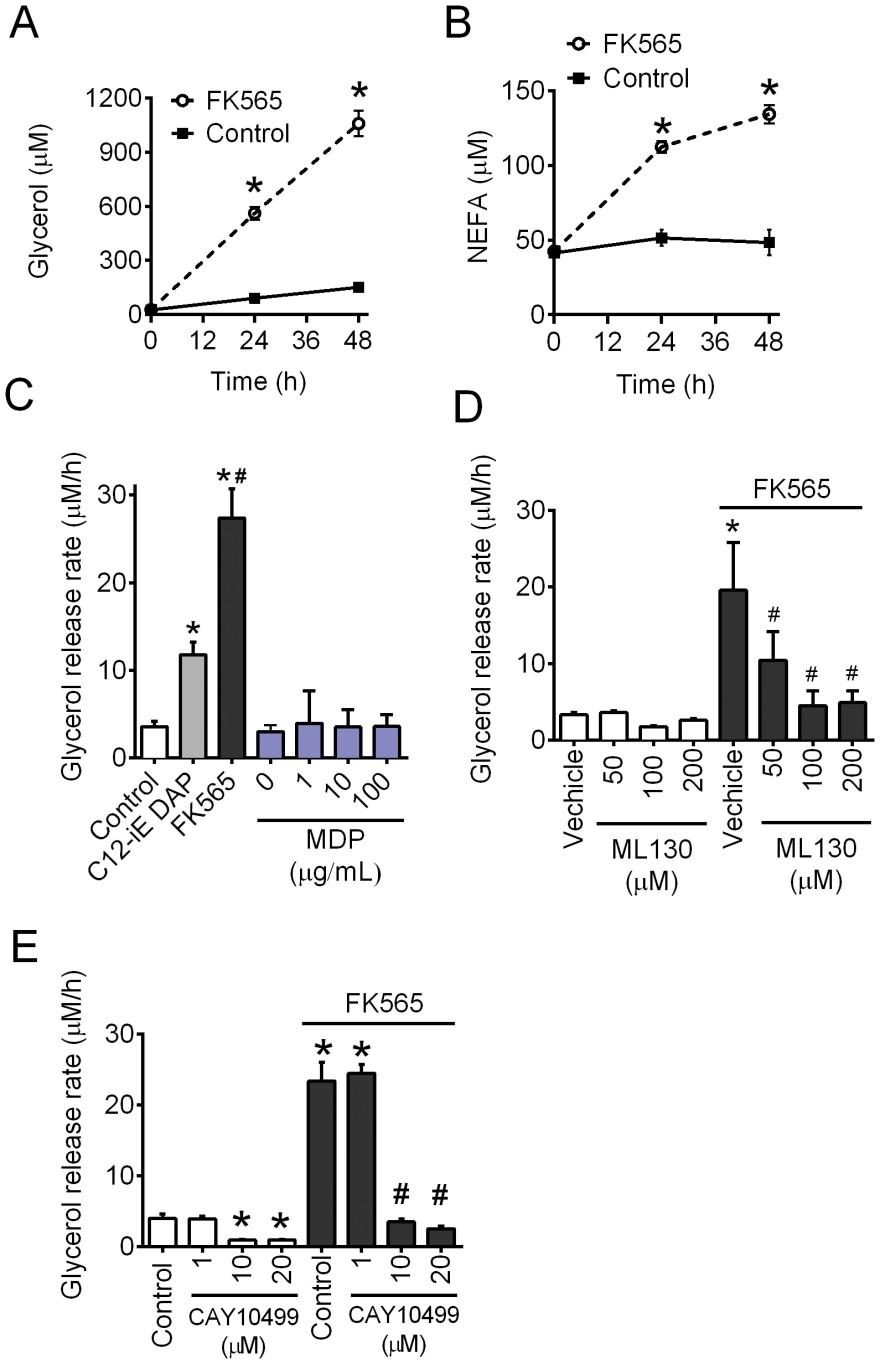
NOD1-activating PGN causes adipocyte-autonomous lipolysis. Differentiated 3T3-L1 adipocytes were incubated with FK565 (10 µg/mL) and glycerol (A) and NEFA (B) concentration in the media determined at 0, 24, 48 h. Glycerol release rate over 48 h was calculated in 3T3-L1 adipocytes, which were incubated with vehicle (control), c12-iEDAP (10 µg/mL), FK565 (10 µg/mL) and various doses of MDP (C). Glycerol release rate over 48 h in 3T3-L1 adipocytes without and with FK565 (10 µg/mL) in the absence or presence of the NOD1 inhibitor ML130 (1-[(4-Methylphenyl)sulfonyl]-1H-benzimidazol-2-amine) at various concentrations (D). Glycerol release rate in 3T3-L1 adipocytes that were incubated with or without FK565 (10 µg/mL) and various concentrations of the HSL inhibitor CAY10499 (E). n = 5–16 experiments per condition, values are mean + SEM. *Significantly different from control or control at a given time point. #Significantly different from c12-iEDAP.

FK565 (10 µg/mL) increased glycerol and NEFA levels released into the media from differentiated 3T3-L1 adipocytes (P<0.05, Fig 2A, B). FK565 increased glycerol release rate more than c12-iEDAP used at the same dose (P<0.05, Fig 2C). The NOD2 ligand, MDP, did not stimulate lipolysis in 3T3-L1 adipocytes (Fig 2C). The recently described NOD1 inhibitor, ML130 (1-[(4-Methylphenyl)sulfonyl]-1H-benzimidazol-2-amine) [31]–[33], completely prevented FK565-induced increases in the rate of glycerol release in 3T3-L1 adipocytes (P<0.05, Fig 2D). FK565-stimulated increases in the rate of glycerol release were also completely prevented with the HSL inhibitor CAY10499 in 3T3-L1 adipocytes (P<0.05, Fig 2E). It is noteworthy that CAY10499 also lowered basal (i.e. unstimulated) glycerol release rate at concentrations (10–20 µM) that block NOD1 ligand mediated lipolysis (Fig. 2E) We next examined what signals are required for NOD1-mediated lipolysis in adipocytes.

### NOD1-activating PGN induces MAPK and PKA signals in adipocytes

**Figure 3:**
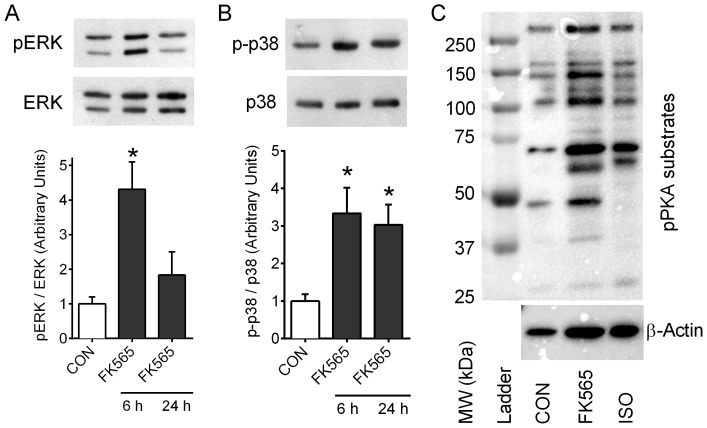
NOD1-activating PGN induces ERK and PKA substrate signals in adipocytes. Differentiated 3T3-L1 adipocytes were incubated with or without FK565 (10 µg/mL) for 6 and 24 h and phosphorylated ERK1/2^Thr202/Tyr204^ (pERK) relative to total ERK (A) and phosphorylated p38 relative to total p38 (B) protein levels were determined in cell lysates. 3T3-L1 adipocytes were incubated with or without FK565 (10 µg/mL, 48 h) or with isoproterenol (ISO, 1 µM, 30 min) and serine/threonine phosphorylated PKA substrates (consensus sequence: RRXS/T) were probed in cell lysates (C). Phospho-PKA substrates immunoblot is indicative of four independent replicates and protein loading was measured with β-Actin immunoblotting (C). n = 4–6 experiments per condition, values are mean + SEM. *Significantly different from control.

NOD1 activation transiently increased the phosphorylation of ERK (pERK). FK565 (10 µg/mL) increased pERK relative to total ERK by ∼3–4 fold at 6 h (P<0.05) in 3T3-L1 adipocytes, an effect that was absent after 24 h (Fig 3A). NOD1 also activated another MAPK, p38 in 3T3-L1 adipocytes, since p-p38 relative to total p38 was increased by ∼3 fold at 6 h and 24 h after FK565 exposure (P<0.05, Fig 3B). We found no evidence that FK565 changed the phosphorylation status of JNK in 3T3-L1 adipocytes (data not shown). Similar to 30 min of isoproterenol, FK565 (48 h, 10 µg/mL) also increased the phosphorylation of specific PKA substrates in 3T3-L1 adipocytes (Fig 3B). The identities of these PKA substrates are not yet known. We next examined if ERK or PKA signaling mechanisms were required for NOD1-mediated lipolysis.

### NOD1-mediated lipolysis occurs via both ERK and PKA

**Figure 4:**
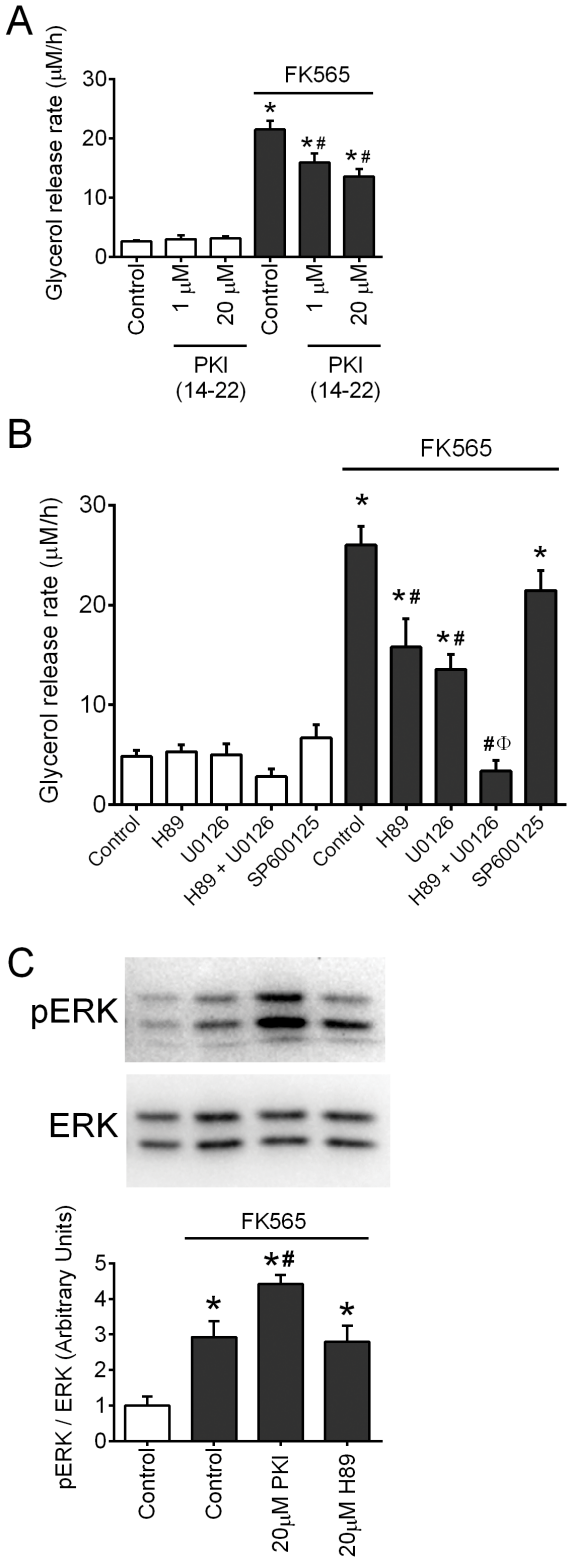
NOD1-mediated lipolysis occurs via both ERK and PKA. Glycerol release rate over 48-L1 adipocytes, which were incubated without or with FK565 (10 µg/mL) and various concentrations of the PKA inhibitor, PKI (14–22) (A). Glycerol release rate over 48 h was calculated in 3T3-L1 adipocytes, which were incubated without or with FK565 (10 µg/mL) and various combinations of H89 (20 µM, PKA inhibitor), and U0126 (10 µM, ERK1/2 inhibitor) or SP600125 (20 µM, JNK inhibitor) (B). 3T3-L1 adipocytes were incubated with or without FK565 (10 µg/mL) for 6 h and phosphorylated ERK1/2^Thr202/Tyr204^ (pERK) relative to total ERK was determined in the presence or absence of the PKA inhibitors PKI and H89 (C). n = 6–16 experiments per condition, values are mean + SEM. *Significantly different from control without FK565. #Significantly different from control with FK565. φSignificantly different from H89 or U0126 alone with FK565.

FK565-stimulated glycerol release rate from 3T3-L1 adipocytes was attenuated ∼35% by PKI (14–22), a peptide-based inhibitor of PKA (P<0.05, Fig 4A). Similarly, the PKA inhibitor H89 only partially attenuated FK565-stimulated glycerol release rate from 3T3-L1 adipocytes (P<0.05, Fig 4B). The MEK1/2 inhibitor (U0126), which effectively blocks ERK activation, also only partially attenuated FK565-stimulated glycerol release rate from 3T3-L1 adipocytes (P<0.05, Fig 4B). However, the combination of both PKA inhibition (H89) and ERK inhibition (U0126) completely prevented FK565-stimulated glycerol release rate from 3T3-L1 adipocytes (P<0.05, Fig 4B). The JNK inhibitor, SP600125 had no effect on FK565-stimulated glycerol release rate from 3T3-L1 adipocytes (Fig 4B). We also showed that the PKA inhibition did not decrease FK565-stimulated pERK in 3T3-L1 adipocytes (Fig 4C). In fact, PKI augmented pERK during FK565 exposure. Our results show that NOD1-activating PGN engages a lipolytic program that involves both ERK and PKA pathways, which appear to converge on HSL to augment lipolysis in adipocytes. We next examined the involvement of NF-κB.

### NOD1-mediated lipolysis engages NF-κB

**Figure 5:**
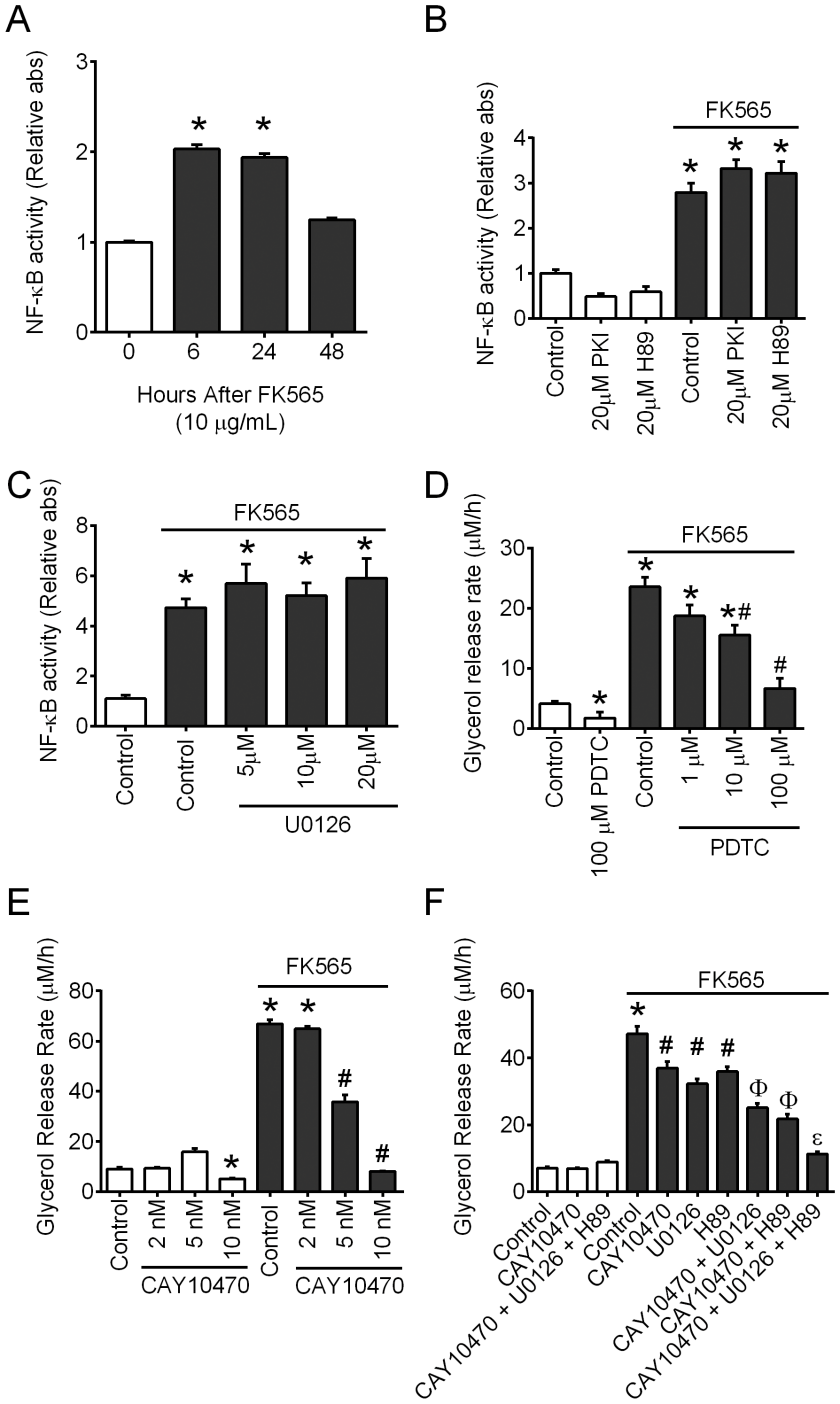
NF-κB is involved in NOD1-mediated lipolysis. NF-κB activity in 3T3-L1 adipocytes after various exposure times of FK565 (10 µg/mL) (A). NF-κB activity in 3T3-L1 adipocytes without and with 6h exposure to FK565 (10 µg/mL) in the presence and absence of PKI (20 µM) and H89 (20 µM) (B). NF-κB activity in 3T3-L1 adipocytes without and with 6h exposure to FK565 (10 µg/mL) with various doses of U0126 (C). n = 4-6 experiments per condition for NF-κB activity. Glycerol release rate over 48 h in 3T3-L1 adipocytes incubated without or with FK565 (10 µg/mL) with various concentrations of NF-κB inhibitors, PDTC (D) and CAY10470 (E). Glycerol release rate over 48 h in 3T3-L1 adipocytes incubated without or with FK565 (10 µg/mL) with various combinations of H89 (20 µM, PKA inhibitor), and U0126 (20 µM, ERK1/2 inhibitor) or CAY10470 (NF-κB inhibitor, 5 nM) (F). n = 12–24 experiments per condition of lipolysis. Values are mean + SEM. *Significantly different from control without FK565. #Significantly different from control with FK565. φSignificantly different from CAY10470 or U0126 or H89 alone with FK565. εSignificantly different from CAY10470 plus U0126 or CAY10470 plus H89 with FK565.

NOD1 activation with FK565 transiently increased NF-kB activity in 3T3-L1 adipocytes at 6 and 24 hours after ligand exposure (P<0.05, Fig 5A). Inhibition of PKA with PKI or H89 did not suppress NF-κB activation by FK565 in 3T3-L1 adipocytes (Fig 5B). Similarly, inhibition of ERK with various doses of U0126 also did not alter NOD1-mediated NF-κB activation (Fig 5C). The NF-κB inhibitor PDTC, when used at 10 µM, partially attenuated FK565-stimulated glycerol release rate from 3T3-L1 adipocytes (P<0.05, Fig 5D). Similarly, the NF-κB inhibitor CAY10470, when used at 5 nM, partially attenuated FK565-stimulated glycerol release rate from 3T3-L1 adipocytes (P<0.05, Fig 5E). These results indicate that NF-κB is involved in at least part of the lipolytic program induced by NOD1 activation in adipocytes. When used at the highest doses both PDTC (100 µM) and CAY10470 (10 nM) inhibited basal lipolysis and prevented FK565-stimulated glycerol release rate from 3T3-L1 adipocytes (Fig 5C, D). Importantly, we also showed that inhibiting NF-κB with CAY10470 (5 nM, a dose that does not alter basal lipolysis) in combination with either ERK or PKA inhibition significantly suppressed FK565-stimulated lipolysis (Fig 5E). This is important because it shows that the partial inhibitory effects on these pathways are additive and that ERK, PKA and NF-κB all play a role in NOD1-mediated lipolysis. Inhibiting all 3 pathways (ERK, PKA and NF-κB) completely prevented NOD1-mediated lipolysis, which is similar to combining ERK and PKA inhibitors (Figure 4B).

## Discussion

Inflammation drives some of the metabolic defects that contribute to obesity-induced disease. Hence, it is important to understand specific metabolic processes that are altered by potential inflammatory triggers and the host receptors that dictate these interactions in metabolic tissues. We previously showed that bacterial PGN can cause whole body and muscle insulin resistance via NOD receptors [20], [23]. We and others also showed previously that NOD1 ligands cause inflammation and impaired insulin action in adipocytes [22]. We now show that NOD1-activating PGN causes lipolysis in adipocytes.

Using knockout mice, our results show that NOD1 is absolutely required for meso-DAP containing PGN motifs to stimulate glycerol and free fatty acid release from adipose tissue. The delineation of the ligand-receptor interaction required for lipolysis is the most important finding in this study, since it demonstrates independence of this lipolytic response from other inflammatory or metabolic pathways. This is an important finding because mammals have a complex system to detect PGN, which includes NOD proteins, PGN recognition proteins, and enzymes capable of processing PGN [34], [35]. We show that NOD1, but not NOD2 ligands stimulate lipolysis and demonstrate an adipocyte cell autonomous NOD1-mediated lipolytic response. Adipose tissue has a plethora of non-adipocyte cells that could potentially mediate inflammation-driven lipolysis and our data shows a direct action of NOD1 on lipolysis in adipocytes. Our data bolsters the concept that the NOD1-activating PGN characteristic of Gram negative bacteria results in more deleterious metabolic effects compared to MDP, which activates NOD2 [15], [21].

We show that NOD1-activating PGN induces MAPK, NF-κB, and PKA signals in adipocytes. Activation of the stress kinase, ERK, by NOD1 has been reported in other cell types [36]. PGN-stimulated phosphorylation of ERK in adipocytes is similar to the effect of other bacterial components such as LPS [27]. Surprisingly, we have shown that NOD1-activating PGN induces the phosphorylation of PKA substrates. It is an important future task to identify these proteins and confirm that these substrates are NOD1-dependent. These exciting results prompted us to examine whether a NOD1-PKA pathway regulated PGN-stimulated lipolysis. Importantly, we tested both chemical and peptide-mediated inhibition of PKA. Results obtained using only chemical inhibitors of PKA (such as H89) are confounded by the potential off target effects of H89 on AMPK, ERK and PKCs [37]. Nevertheless, PKA inhibition only partially inhibited NOD1-mediated lipolysis in 3T3-L1 adipocytes. Similarly, ERK inhibition only partially inhibited NOD1-mediated lipolysis. It appears that NOD1 engages both ERK and PKA pathways to regulate lipolysis, since simultaneously inhibiting both of these pathways completely prevented any increase in glycerol release from adipocytes. ERK is a well-known factor in the NF-κB pathway and others have shown connections between PKA and NF-κB signaling events, even in response to inflammatory triggers such as TNFα [38], [39]. Intriguingly, PKA subunits can be found in an NF-κB and IκB complex. Given this interaction, signals the degrade IκB can activate PKA and PKA can actually regulate the phosphorylation of NF-κB thereby altering its transcriptional activity [38]. It is not entirely clear how PKA and NF-κB interact in the adipocyte, but our results show that NOD1 engages both of these signaling pathways to augment lipolysis.

There is still a lack of consensus regarding the signaling cascades engaged by inflammatory, lipolytic triggers. For example, some groups show the importance of solely ERK [27] and others showing that both ERK and PKA are required for LPS-stimulated lipolysis in adipocytes [40]. We sought to determine if NOD1-stimulated ERK or PKA signals also engaged NF-κB. We found NOD1 activation of NF-κB was not changed by inhibiting PKA or ERK (when measured by p65 binding to an NF-κB DNA consensus sequence). NOD1-mediated lipolysis engaged NF-κB, since several inhibitors of this pathway attenuated PGN-stimulated lipolysis in a manner that was additive with either PKA or ERK pathways. Our results show that simultaneously inhibiting any two pathways of PKA, NF-κB and ERK prevents NOD1-mediated lipolysis in adipocytes. However, we have not yet addressed the role of specific cytokines in NOD1-mediated lipolysis. Type 1 interferon signaling can dominate over the NF-κB pathway in human epithelial cell lines [41]. Hence, it is an important future goal to understand the involvement of interferon response in NOD1-mediated lipolysis in adipocytes. This is may be relevant because interferon alpha and interferon gamma can stimulate adipocyte lipolysis [42].

**Figure 6:**
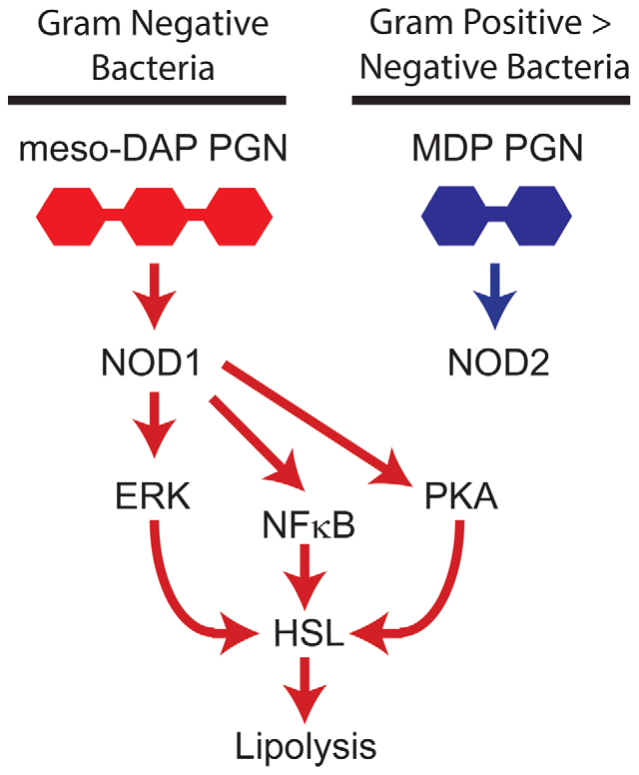
Working model of bacterial PGN-mediated lipolysis in adipocytes. The bacterial peptidoglycan motifs that activate NOD1cause cell autonomous lipolysis in adipocytes. NOD1-activating PGN engages ERK, PKA and NF-κB signals leading to lipolysis. NOD1-mediated signals converge on HSL to stimulate adipocyte lipolysis. Our evidence suggests that separate engagement of ERK and PKA pathways are needed to fully activate NOD1-mediated lipolysis in adipocytes.

This work has built a picture of how adipocyte lipolysis is stimulated by bacterial cell wall components (Fig 6). We show that HSL is a lipase involved in the NOD1-stimulated lipolytic program in adipocytes. However, it is not yet entirely clear how ERK, PKA, and NF-κB conspire through various lipases and perilipin to promote lipolysis in response to bacterial triggers. Others have recently confirmed that Tri-DAP-based PGN ligands cause a relatively small increase in adipocyte lipolysis and they identified some of the potential HSL phosphorylation (i.e. serine 563) sites regulated by NOD1 through PKA and NF-κB[43]. Our results build on this recent result by implicating separate inputs from PKA, ERK and NF-κB in the regulation of NOD1-mediated lipolysis in adipocytes. Importantly, we have shown that the type of PGN generally found in gram negative bacteria acts on NOD1 to augment lipolysis in adipose tissue.

In summary, we show that bacterial PGN stimulates NOD1-dependent adipocyte lipolysis by engaging stress kinase, PKA and NF-kB pathways. This work has uncovered an important metabolic defect induced by a specific component of the bacterial cell wall. Gram-negative bacteria generally contain both LPS and NOD1-activating PGN and now both of these bacterial components are known to promote lipolysis in adipocytes. It is important to understand the triggers and pathways of adipocyte lipolysis because modifying HSL-mediated WAT lipolysis alters whole body insulin and glucose tolerance [44]. We propose that NOD1-activating PGN contributes to lipolysis and insulin resistance during metabolic endotoxemia.

## Materials and Methods

### Animals

Ethics statement: All protocols, which involved the handling of animals, followed the Canadian Council on Animal Care (CCAC) guidelines and were approved by McMaster University's Animal Research Ethics Board (AREB). C57BL/6 mice were purchased from JAX (Bar Harbor, ME) and NOD1-deficient mice (Millenium Pharmaceuticals, Cambridge, MA) have been backcrossed to C57BL/6 strain for at least 10 generations.

### Materials

FK565 (heptanoyl-γ-D-glutamyl-L-*meso* –diamino-pimelyl-D-alanine) was obtained from Fujisawa Pharmaceuticals (Osaka, Japan). H89, SP600125, U0126, pERK (#4370), ERK (#4695), p-p38 (#9215), p38 (#9212) β-Actin (#5125) and phospho-PKA substrate (#9624) antibodies were from Cell Signaling Technology (Boston, MA, USA). Fatty acid-free bovine serum albumin (BSA), ammonium pyrrolidinedithiocarbamate (PDTC) and isoproterenol were from Sigma Aldrich (St. Louis, MO). CAY10499 and CAY10470 were from Cayman Chemicals (Ann Arbor, MI). Myristoylated PKI (14–22) amide and ML130 were from Tocris Bioscience (Bristol, UK). Dulbecco's modified Eagle medium (DMEM), Dulbecco's Phosphate-Buffered Saline (DPBS) and fetal bovine serum (FBS) were from Life Technologies (Burlington, ON, CA). c12-iEDAP and MDP were from Invivogen (San Diego, CA).

### White adipose tissue explants

Gonadal fat pads were surgically removed from wild-type and NOD1^−/−^ mice and subsequently washed in a pre-warmed solution of phosphate buffered saline (PBS) and 1% penicillin-streptomycin (p/s). The fat pads were then minced into approximately 5 mg pieces and incubated in 10 mL of warmed DMEM supplemented with 10% fetal bovine serum (FBS) and 1% p/s for two h. The adipose tissue pieces were divided into 24-well plates containing 1 mL of DMEM supplemented with 1% fatty acid free BSA and 1% of p/s (approx. 5 mg/well). The explants were subsequently treated with the appropriate ligand and incubated for up to 72 h at 37°C and 5% CO_2_.

### Cell culture

Murine 3T3-L1 pre-adipocytes (American Type Culture Collection, Rockville, MD, USA) were cultured in DMEM supplemented with 10% FBS, 1% glutamine and 1% p/s (culture media). The cells were maintained in an incubator at 37°C and 5% CO_2_. When the cells reached confluence, differentiation was induced by incubating the cells with culture medium containing 0.5 mM 3-isobutyl-1-methylxanthine, 0.25 µM dexamethasone and 10 µg/ml insulin for 48 hours (from day 0 to day 2). After 48 hours, the medium was changed to culture medium containing only 10 µg/ml insulin for 48 hours (from day 2 to day 4) and changed again to culture medium containing only 10 µg/ml insulin for another 48 hours (from day 4 to day 6). Subsequently, the medium was replaced by culture media every 2 days until full differentiation was achieved. Experiments were performed on the differentiated adipocytes between day 8 and 12.

### Experimental conditions

WAT explants were treated with various doses of FK565 or other ligands and media were removed at 0, 24, 48 and 72 hours. All subsequent experiments were performed using 10 µg/ml of FK565. The incubation media were removed at 0, 6, 24 and/or 48 hours of treatment in differentiated 3T3-L1 adipocytes. Prior to treatment, the 3T3-L1 adipocytes were washed twice with serum-free DMEM and cultured in medium containing serum-free DMEM, 0.5% fatty acid-free BSA and 1% p/s. The adipocytes were pre-incubated for 10 min, 30 min or 1 h with the inhibitors and subsequently treated with or without 10 µg/ml of FK565. Lipolysis was assessed *in vitro* by measuring the concentration of glycerol and/or NEFA released into the supernatant media. Glycerol concentrations were determined by using a free glycerol determination kit from Sigma-Aldrich (St. Louis, MO, USA). NEFA concentrations were determined using an enzymatic colorimetric method assay from Wako Diagnostics (Richmond, VA, USA). In experiments using WAT explants, the glycerol and NEFA concentrations were corrected to tissue weight. For protein analysis, 3T3-L1 adipocytes were lysed in ice-cold lysis buffer containing 250 mM NaCl, 50 mM NaF, 5 mM EDTA, 1% triton X-100 and 50 mM Tris-HCl (pH 7.4). Protein concentration was measured using a bicinchoninic acid (BCA) assay kit (Pierce, Rockford, IL, USA). Western blotting was performed as described previously [45], [46]. Briefly, lysates were denatured, separated by SDS-PAGE, transferred to polyvinylidene fluoride (PVDF) membranes. Membranes were blocked and immunoblotted with ERK (1∶2000), phospho-ERK (1∶2000), β-actin (1∶2000) and phospho-PKA substrate (1∶2000) antibodies. Lysates were also used to determine NF-κB activity using a commercially available DNA binding ELISA (#40096, Active Motif, Carlsbad, CA, USA).

### Data analysis

Data are expressed as mean ± standard error of the mean (SEM). Comparisons were made using two-tailed Student's t test or ANOVA, where appropriate. Differences were considered statistically significant at P<0.05. The rate of glycerol release was determined by taking the slope of the linear fit of the cumulative glycerol release over the 48 h or 72 h incubation period.
